# Proteostasis and ageing: insights from long‐lived mutant mice

**DOI:** 10.1113/JP274334

**Published:** 2017-08-02

**Authors:** William A. Sands, Melissa M. Page, Colin Selman

**Affiliations:** ^1^ Glasgow Ageing Research Network (GARNER), Institute of Biodiversity, Animal Health and Comparative Medicine, College of Medical, Veterinary and Life Sciences University of Glasgow Glasgow G12 8QQ UK; ^2^ Department of Cellular and Physiological Sciences University of British Columbia Vancouver BC V6T 1Z3 Canada

**Keywords:** ageing, endoplasmin reticulum stress, immunoproteasome, longevity, proteasome, proteostasis, unfolded protein response

## Abstract

The global increase in life expectancy is creating significant medical, social and economic challenges to current and future generations. Consequently, there is a need to identify the fundamental mechanisms underlying the ageing process. This knowledge should help develop realistic interventions capable of combatting age‐related disease, and thus improving late‐life health and vitality. While several mechanisms have been proposed as conserved lifespan determinants, the loss of proteostasis – where proteostasis is defined here as the maintenance of the proteome – appears highly relevant to both ageing and disease. Several studies have shown that multiple proteostatic mechanisms, including the endoplasmic reticulum (ER)‐induced unfolded protein response (UPR), the ubiquitin–proteasome system (UPS) and autophagy, appear indispensable for longevity in many long‐lived invertebrate mutants. Similarly, interspecific comparisons suggest that proteostasis may be an important lifespan determinant in vertebrates. Over the last 20 years a number of long‐lived mouse mutants have been described, many of which carry single‐gene mutations within the growth‐hormone, insulin/IGF‐1 or mTOR signalling pathways. However, we still do not know how these mutations act mechanistically to increase lifespan and healthspan, and accordingly whether mechanistic commonality occurs between different mutants. Recent evidence supports the premise that the successful maintenance of the proteome during ageing may be linked to the increased lifespan and healthspan of long‐lived mouse mutants.

**Abbreviations**
DR: dietary restriction
ERAD: endoplasmic reticulum‐associated degradation
GH: growth hormone
IGF‐1: insulin‐like growth factor‐1
IIS: insulin/IGF‐1 signalling
mTOR: mechanistic (mammalian) target of rapamycin
UPR^ER^: endoplasmic reticulum unfolded protein response
UPR^mt^: mitochondrial unfolded protein response
UPS: ubiquitin‐proteasome system

## Introduction

It is now well established that the changes that occur during ageing at the level of the phenotype in multicellular organisms is highly conserved, and that ageing rate can be modulated through a number of environmental, genetic and pharmacological means (Gems & Partridge, 2013; Lamming *et al*. 2013; Selman, 2014). Over the last couple of decades a significant number of studies have demonstrated that disrupting signalling through the growth hormone (GH) (Brown‐Borg *et al*. 1996; Coschigano *et al*. 2003), the insulin/IGF‐1 (Selman *et al*. 2008) and the mechanistic target of rapamycin (mTOR) pathways (Selman *et al*. 2009; Arif *et al*. 2017) can extend lifespan in mice and improve late‐life health. Excitingly, pharmacological manipulation of some of these pathways can also slow ageing in mice (Harrison *et al*. 2009), and polymorphisms in genes within these pathways are correlated with human longevity (Deelen *et al*. 2013; Passtoors *et al*. 2013). Without doubt one of the greatest challenges in mouse ageing research currently is to try and identify mechanistically how these interventions act to elicit their favourable effects, and to determine whether shared mechanisms drive longevity and healthspan across different mutants or whether such mechanisms are only specific to a particular mutant or specific signalling pathway. Such information is likely to be crucial if we are to ultimately design safe and effective interventions to extend late‐life health and vitality in humans.

The successful maintenance of proteins within cells is termed proteostasis, which involves a number of cellular processes encompassing the initial synthesis of nascent proteins, through to the appropriate folding, transport and secretion of mature proteins, to the degradation (and recycling) of damaged and redundant proteins within the cell. Consequently, the inability of the cellular proteostatic machinery to maintain the proteome appropriately over the life‐course has been implicated heavily in the ageing process and in underlying a number of age‐associated pathologies (Vilchez *et al*. 2014; Labbadia & Morimoto, 2015). Cells contain a number of well‐described mechanisms to help maintain proteostasis: the unfolded protein response (UPR), which is initiated following accumulation of unfolded/misfolded proteins within the endoplasmic reticulum (UPR^ER^) or following mitonuclear protein imbalance and mitochondrial dysfunction (UPR^mt^), the ubiquitin–proteasome system (UPS) and the lysosomal–autophagy pathway. In this brief review we will specifically focus on three components of the cellular proteostatic machinery, namely the UPR^ER^ and UPR^mt^, and the UPS, and document the current evidence linking longevity in mutant mice to these proteostatic processes. Due to space issues we will not discuss the lysosomal–autophagy pathway, but direct the reader to excellent recent reviews on this subject (Lapierre *et al*. 2015; Carmona‐Gutierrez *et al*. 2016).

## Endoplasmic reticulum (ER) stress and the unfolded protein response (UPR)

**Figure 1:**
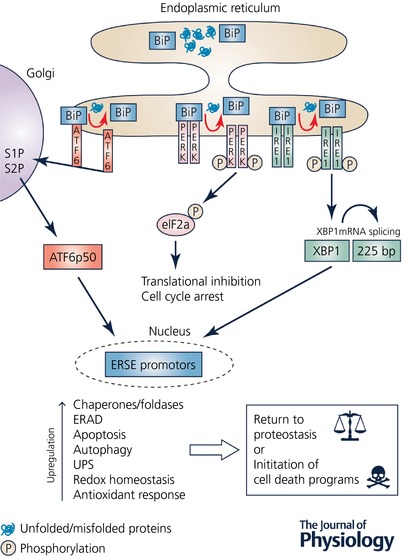
Main components of the endoplasmic reticulum (ER) unfolded protein response (UPR) in mammals The intracellular build‐up of unfolded/misfolded proteins results in ER stress that initiates an adaptive response – the UPR – which invokes a cellular cascade in an attempt to return the cell back to proteostasis. The proximal UPR sensors are PKR‐like ER kinase (PERK), inositol requiring element‐1 (IRE1), and activating transcription factor 6 (ATF6). Following ER stress, BiP/GRP78 disassociates from PERK, IRE1 and ATF6, resulting in the initiation of the UPR, and also allows *BiP* itsel to undertake various chaperone activities. Activated PERK results in the phosphorylation of eukaryotic initiation factor 2α (eIF2α) that leads to inhibition of translation and cell cycle arrest, thus reducing protein loading within the cell. IRE1 splices a 225 base pair intron from its substrate XBP1, thus activating XBP1, which translocates to the nucleus and binds to specific endoplasmic reticulum stress elements (ERSE) within the nucleus resulting in the upregulation in expression of multiple genes, including cellular chaperones and foldases. Upon disassociation from Bip, ATF6 is cleaved by site‐1 protease (S1P) and site‐2 protease (S2P) within the Golgi apparatus to an active form ATF6 p50, which then translocates to the nucleus and induces endoplasmic reticulum associated protein degradation (ERAD) by the ubiquitin–proteasome system (UPS). If the ER stress is prolonged or severe and the UPR cannot return the cell to proteostasis, then cell death programmes, including the apoptosis cascade, will be initiated in order to remove the damaged cells.

Proteostatic stress, through the build‐up of aberrant (misfolded or unfolded) proteins can initiate a coordinated stress response within the ER – the UPR^ER^ (Fig. 1). During ER stress, the UPR instigates a triad of adaptive cellular responses to reduce protein loading and initiate a return to proteostasis; (i) a general reduction in protein synthesis through translational repression,( ii) an upregulation in specific chaperones and foldases to increase protein folding capacity, and (iii) an enhancement in ER‐associated degradation (ERAD) of aberrant proteins by the proteasome (Labbadia & Morimoto, 2015). The proximal sensors of the UPR are inositol requiring element‐1 (IRE1), PKR‐like ER kinase (PERK) and activating transcription factor 6 (ATF6). Upon ER stress, BiP/GRP78, a HSP70 family member, disassociates from these three sensors, thus triggering the UPR and enabling BiP/GRP78 to undertake chaperone activities in response to the accumulation of misfolded and unfolded proteins (Labbadia & Morimoto, 2015).

Several elements of the UPR machinery show a general decline in activity with advancing age (Naidoo, 2009), with various chaperones showing significant age‐associated reductions at both the mRNA and protein level in mice (Nuss *et al*. 2008). In *C. elegans*, the induction of a number of components of the UPR^ER^ following tunicamycin treatment decreased with advancing age, including transcript levels of spliced *xbp‐1* and UPR^ER^ target genes (Taylor & Dillin, 2013). Constitutive activation of *xbp‐1* was sufficient to increase late‐life resistance to ER stress but did not affect worm lifespan (Taylor & Dillin, 2013). Interestingly, these same authors then went on to show that both neuronal‐ and intestinal‐specific activation of *xbp‐1* increased lifespan in *C. elegans*, and that neuronal activation both induced the UPR^ER^ within intestinal cells and rescued ER stress resistance during ageing (Taylor & Dillin, 2013). Inactivation of specific UPR genes shortened the lifespan of long‐lived mutant worms (Henis‐Korenblit *et al*. 2010; Shore *et al*. 2012), and intestinal IRE1 appears necessary for lifespan extension under dietary restriction (DR) in *Drosophila* (Luis *et al*. 2016). In addition, DR appears to afford protection against age‐associated declines in components of the proteostatic network in mouse liver (Mitchell *et al*. 2016). Several comparative studies have investigated components of the UPR^ER^, with longer‐lived species tending to have greater constitutive levels of chaperones (e.g. HSP60, HSP70, HSP90, GRP78) compared to shorter‐lived species (Salway *et al*. 2011a; Pride *et al*. 2015). However, in long‐lived Snell and *Ghr^−/−^* mice, mRNA transcript levels of various chaperones did not show a consistent increase in expression across a range of tissues (Swindell *et al*. 2009).

Almost without exception, the little research examining proteostasis in long‐lived mutant mice has been undertaken in long‐lived GH‐deficient Snell dwarf mice. Primary skin fibroblasts from Snell dwarfs have been shown to be hypersensitive to the ER stressors thapsigargin and tunicamycin compared to cells derived from control mice (Salmon *et al*. 2008). In agreement, fibroblasts derived from long‐lived naked mole rats (Salmon *et al*. 2008) and long‐lived IIS mutant mice (M. M. Page, D. J. Withers and C. Selman, unpublished observations) are similarly significantly more sensitive to ER stress than cells derived from wild‐type mice. The ER‐stress sensitive phenotype of Snell dwarf fibroblasts was associated with lower *Ire1a* expression, a reduced ratio of spliced:unspliced *Xbp1* and a lower expression of various *Xbp1*‐associated target genes (Sadighi Akha *et al*. 2011). While ER stress did not differentially affect several chaperones (BiP, PDI, ERp72, GRP94) in Snell dwarf fibroblasts compared to control fibroblasts, various pro‐apoptotic markers (e.g. CHOP, caspase‐12 levels, caspase‐3 activity, c‐JUN phosphorylation) were all significantly increased in Snell dwarf fibroblasts following ER stress (Sadighi Akha *et al*. 2011). These findings led the authors to speculate that the ER stress sensitive phenotype of Snell dwarf fibroblasts enabled a more rapid induction of apoptosis and subsequent removal of damaged cells, leading to a quicker return to proteostasis. Interestingly, IRE1 activation in human brain correlates with Alzheimer's disease (AD) pathology, and neuronal‐specific deletion of *Ire1* in a mouse model of AD reduced amyloid precursor protein expression and rescued learning and memory capacity (Duran‐Aniotz *et al*. 2017). The transcription factor ATF4, a central component of the cellular stress response, and several of its downstream targets are also elevated in fibroblasts from both long‐lived Snell and PAPP‐A knockout mice following tunicamycin exposure (Li & Miller, 2015). Hepatic ATF4 protein levels are also significantly increased in a number of different long‐lived mouse models (Li *et al*. 2014). While clearly more research needs to be done in other mutant models, it has been shown that the addition of IGF1 to murine NIH/3T3 cells previously exposed to ER stress causes attenuation of apoptosis and an induction in *BiP/Grp78* expression (Novosyadlyy *et al*. 2008). Reducing IIS pharmacologically in cells through NT219 treatment, which impairs IGF1R kinase activity and causes degradation of insulin receptor substrates 1 and 2, activated various chaperones, leading to the accrual of prion protein within intracellular aggresomes, although interestingly NT219 reduced both proteasomal and autophagy activity (Moll *et al*. 2016).

The mitochondrial unfolded protein response (UPR^mt^) is a highly conserved signalling response, which is induced following mitonuclear protein imbalance and dysfunction, leading to a cytoprotective transcriptional response that maintains proteostasis and has been reported to correlate with lifespan (Jovaisaite & Auwerx, 2015). A number of studies have identified associations between the induction of the UPR^mt^ and longevity in worms (Rauthan *et al*. 2015; Merkwirth *et al*. 2016), flies (Owusu‐Ansah *et al*. 2013) and mice (Houtkooper *et al*. 2013; Merkwirth *et al*. 2016), and UPR^mt^ may help explain the longevity of mitochondrial mutants (Lapointe & Hekimi, 2008; Ristow & Schmeisser, 2011). However, it should also be noted that other studies have reported no such association (Bennett *et al*. 2014; Mulvey *et al*. 2016), and indeed UPR^mt^ induction may be detrimental under certain conditions (Lin *et al*. 2016). Surprisingly little research on the UPR^mt^ has been undertaken in long‐lived mutant mice, although markers of the UPR^mt^ were recently shown to be elevated in cells and tissues from long‐lived Surf1 knockout mice (Pharaoh *et al*. 2016).

## The ubiquitin–proteasome system

The ubiquitin–proteasome system (UPS) plays a critical role in maintaining proteostasis within cells through the recognition and subsequent degradation of misfolded and damaged proteins, and by maintaining quality control of newly synthesised proteins. The UPS is a highly conserved proteolytic pathway that consists of a core 20S catalytic particle, whose activity is subsequently modulated by a number of regulatory subunits including the 11S (PA28) and 19S (PA700) subunits (Jung & Grune, 2012). Interaction of the 19S regulator with two α‐rings on the 20S core, leads to the formation of a larger 26S proteasome complex that accounts for the majority of the proteolytic activity within cells under steady‐state conditions (Jung & Grune, 2012). An inducible form of the 20S proteasome (i20S), induced by cytokines such as interferon gamma (IFNɣ) and tumour necrosis factor alpha (TNFα), also exists, which is thought to be key to antigen presentation by the major histocompatibility complex (Johnston‐Carey *et al*. 2015). In common with the other proteostatic pathways, multiple components of the UPS are known to be negatively affected by ageing (Grune *et al*. 2001; Vilchez *et al*. 2014). For example, activity of the 26S proteasome declines in human peripheral blood lymphocytes with age, and this was correlated with an increased number of post‐translational modifications on proteasomal subunits (Carrard *et al*. 2003). Similar declines in the activity of both the 20S and 26S proteasome have been described during ageing and senescence in a wide‐range of species, tissues and cells (Vilchez *et al*. 2014; Raynes *et al*. 2017).

Elevated UPS activity has been correlated with longevity in model organisms (Tonoki *et al*. 2009; Kruegel *et al*. 2011; Rana *et al*. 2013; Chondrogianni *et al*. 2015), and appears essential to the longevity of IIS mutant worms (Ghazi *et al*. 2007; Matilainen *et al*. 2013). Proteasomal function is also preserved in cells derived from human centenarians (Chondrogianni *et al*. 2000), and intranasal administration of human HSP70 to aged mice increased proteasome activity within the cerebral cortex, improved cognitive function and extended lifespan relative to untreated mice (Bobkova *et al*. 2015) In contrast, impairing the UPS shortens lifespan and increases age‐associated pathology in flies (Liu & Pfleger, 2013) and mice (Min *et al*. 2008), and UPS dysfunction is associated with a number of human diseases (McKinnon & Tabrizi, 2014). Using an orthologue mapping approach it has also been shown that proteins associated with the UPS are candidate targets of selection in mammalian lineages associated with longevity (Li & de Magalhaes, 2013). Additional studies have reported that UPS activity tends to be enhanced within cells and tissues of long‐lived organisms such as naked mole rats (Pride *et al*. 2015; Rodriguez *et al*. 2016), although these findings are not consistent across all studies (Salway *et al*. 2011b). Recently, it was reported that lifespan in primates was positively correlated with activity of the 20S, but not the 26S, proteasome within primary fibroblasts (Pickering *et al*. 2015). Further examination linked this association specifically to the immunoproteasome, with protein and mRNA levels of the immunoproteasome subunit PSMB8 in primary fibroblasts correlating with longevity across different primate species. Hepatic PSMB8 protein levels were similarly elevated in several long‐lived mouse models, including Snell dwarf mice and rapamycin fed mice (Pickering *et al*. 2015). However, proteasomal activity was not higher in liver (Pickering *et al*. 2015), heart or brain of Snell dwarf mice relative to controls (Salway *et al*. 2011b), and cardiac levels of both the 20S proteasome and the immunoproteasome were reduced in mice treated intraperitoneally with rapamycin for 7 days (Zhang *et al*. 2015). As mentioned previously, reduced insulin/IGF‐1 signalling (IIS) correlates with longevity across model organisms and also in humans (Gems & Partridge, 2013). A novel insight into the dynamic relationship between IIS and proteostasis in the context of ageing was recently provided by Tawo and colleagues (Tawo *et al*. 2017), who showed that the E3 ubiquitin ligase CHIP, a critical regulator of proteostasis, is involved in proteolysis of the insulin receptor, and that this function may promote longevity in worms and flies. Importantly, under conditions of proteostatic stress and during ageing, CHIP is preferentially directed towards the disposal of aberrant proteins and away from targeted degradation of the insulin receptor (Tawo *et al*. 2017).

## Concluding remarks

Despite the relatively large number of long‐lived mutant mice being described over the last couple of decades, there is a clear absence of information regarding whether the underlying mechanism driving these longevity phenotypes show commonality across different mutants or are simply specific to a particular mutant. Such conservation is important in our quest to understand the mechanistic nature of ageing in model organisms and relate this ultimately to human ageing. For example, it is evident that cellular resistance to oxidative stress is not conserved across all long‐lived mutant mice (Page *et al*. 2014; Hofmann *et al*. 2015). It is well established that organisms are endowed with a large number of tools to maintain proteostasis within their cells, and it is unequivocal that the effectiveness of this tool‐kit declines over the life‐course of an organism and that this decline is pervasively implicated in both ageing and age‐related pathology. Emerging evidence is now revealing that various components of the proteostastic network are essential for longevity in some mutant worms and flies, and that the maintenance of these networks during ageing may also underlie lifespan differences across different vertebrates. However, there undoubtedly needs to be an improved appreciation of whether proteostatic mechanisms differ across particular long‐lived mutant mice relative to control mice under both basal and stressed (including ‘natural’ ageing) conditions. Virtually all of what we currently understand about cellular stress phenotypes in long‐lived mutants has been gleaned from studies in primary fibroblasts. There is clearly a need for a greater investigation of cellular stress phenotypes across different cell and tissue types, and whether sex‐specific effects exist, particular relevant to those mutants and interventions that show clear sex‐specific differences in longevity. In addition, a relatively straightforward comparative approach examining proteostasis in different mouse strains that are known to vary in ‘natural’ longevity may also provide additional insights. Given that significant interplay exists between different components of the proteostatic network, with compensation existing between proteasomal function and autophagy for example (Ding *et al*. 2007), it is certainly feasible that different mouse mutants may employ subtlety different ways in which to maintain their proteome, although the ultimate goal of preserving an optimal proteome is shared. A number of conditional mutants of the original long‐lived mutants now exist and it will be interesting in the future to also investigate, as has been done in invertebrate model organisms, proteostatic mechanisms in the context of cell autonomous *versus* non‐autonomous actions (see O'Brien & van Oosten‐Hawle, 2016). As many of the genetic pathways known to extend lifespan in mice are pharmacologically amenable and given that several components of the proteostatic network are also druggable, specific targeting of specific pathways and proteostatic components should help to further drive forward our understanding of the relevance of proteostasis in mammalian longevity and healthspan, and thus help focus research on realistic point of intervention ultimately capable of slowing human ageing and improving late‐life health and wellbeing.

## Additional information

### Competing interests

The authors declare no conflicts of interests.

### Author contributions

All authors were involved in the writing of the manuscript and approved the final version of the manuscript. All individuals designated as authors qualify for authorship, and all individuals who qualify for authorship are listed

### Funding

C.S. acknowledges support from the Biotechnology and Biological Sciences Research Council (BBSRC) through the award of a New Investigator Grant (BB/H012850/1), and from the College of Medical, Veterinary and Life Sciences, University of Glasgow. M.M.P. acknowledges support from the Canadian Diabetes Association through a postdoctoral fellowship.

## References

[tjp12512-bib-0001] Arif A , Terenzi F , Potdar AA , Jia J , Sacks J , China A , Halawani D , Vasu K , Li X , Brown JM , Chen J , Kozma SC , Thomas G & Fox PL (2017). EPRS is a critical mTORC1‐S6K1 effector that influences adiposity in mice. Nature 542, 357–361. 28178239 10.1038/nature21380 PMC5480610

[tjp12512-bib-0002] Bennett CF , Vander Wende H , Simko M , Klum S , Barfield S , Choi H , Pineda VV & Kaeberlein M (2014). Activation of the mitochondrial unfolded protein response does not predict longevity in *Caenorhabditis elegans* . Nat Commun 5, 3483. 24662282 10.1038/ncomms4483 PMC3984390

[tjp12512-bib-0003] Bobkova NV , Evgen'ev M , Garbuz DG , Kulikov AM , Morozov A , Samokhin A , Velmeshev D , Medvinskaya N , Nesterova I , Pollock A & Nudler E (2015). Exogenous Hsp70 delays senescence and improves cognitive function in aging mice. Proc Natl Acad Sci USA 112, 16006–16011. 26668376 10.1073/pnas.1516131112 PMC4702952

[tjp12512-bib-0004] Brown‐Borg HM , Borg KE , Meliska CJ & Bartke A (1996). Dwarf mice and the ageing process. Nature 384, 33. 10.1038/384033a0 8900272

[tjp12512-bib-0005] Carmona‐Gutierrez D , Hughes AL , Madeo F & Ruckenstuhl C (2016). The crucial impact of lysosomes in aging and longevity. Ageing Res Rev 32, 2–12. 27125853 10.1016/j.arr.2016.04.009 PMC5081277

[tjp12512-bib-0006] Carrard G , Dieu M , Raes M , Toussaint O & Friguet B (2003). Impact of ageing on proteasome structure and function in human lymphocytes. Int J Biochem Cell Biol 35, 728–739. 12672464 10.1016/s1357-2725(02)00356-4

[tjp12512-bib-0007] Chondrogianni N , Georgila K , Kourtis N , Tavernarakis N & Gonos ES (2015). 20S proteasome activation promotes life span extension and resistance to proteotoxicity in *Caenorhabditis elegans* . FASEB J 29, 611–622. 25395451 10.1096/fj.14-252189 PMC4314225

[tjp12512-bib-0008] Chondrogianni N , Petropoulos I , Franceschi C , Friguet B & Gonos ES (2000). Fibroblast cultures from healthy centenarians have an active proteasome. Exp Gerontol 35, 721–728. 11053662 10.1016/s0531-5565(00)00137-6

[tjp12512-bib-0009] Coschigano KT , Holland AN , Riders ME , List EO , Flyvbjerg A & Kopchick JJ (2003). Deletion, but not antagonism, of the mouse growth hormone receptor results in severely decreased body weights, insulin, and insulin‐like growth factor I levels and increased life span. Endocrinology 144, 3799–3810. 12933651 10.1210/en.2003-0374

[tjp12512-bib-0010] Deelen J , Uh HW , Monajemi R , van Heemst D , Thijssen PE , Bohringer S , van den Akker EB , de Craen AJ , Rivadeneira F , Uitterlinden AG , Westendorp RG , Goeman JJ , Slagboom PE , Houwing‐Duistermaat JJ & Beekman M (2013). Gene set analysis of GWAS data for human longevity highlights the relevance of the insulin/IGF‐1 signaling and telomere maintenance pathways. Age (Dordr) 35, 235–249. 22113349 10.1007/s11357-011-9340-3 PMC3543749

[tjp12512-bib-0011] Ding WX , Ni HM , Gao W , Yoshimori T , Stolz DB , Ron D & Yin XM (2007). Linking of autophagy to ubiquitin‐proteasome system is important for the regulation of endoplasmic reticulum stress and cell viability. Am J Pathol 171, 513–524. 17620365 10.2353/ajpath.2007.070188 PMC1934546

[tjp12512-bib-0012] Duran‐Aniotz C , Cornejo VH , Espinoza S , Ardiles AO , Medinas DB , Salazar C , Foley A , Gajardo I , Thielen P , Iwawaki T , Scheper W , Soto C , Palacios AG , Hoozemans JJ & Hetz C (2017). IRE1 signaling exacerbates Alzheimer's disease pathogenesis. Acta Neuropathol 134, 489–506. 10.1007/s00401-017-1694-x 28341998

[tjp12512-bib-0013] Gems D & Partridge L (2013). Genetics of longevity in model organisms: debates and paradigm shifts. Annu Rev Physiol 75, 621–644. 23190075 10.1146/annurev-physiol-030212-183712

[tjp12512-bib-0014] Ghazi A , Henis‐Korenblit S & Kenyon C (2007). Regulation of *Caenorhabditis elegans* lifespan by a proteasomal E3 ligase complex. Proc Natl Acad Sci USA 104, 5947–5952. 17392428 10.1073/pnas.0700638104 PMC1851597

[tjp12512-bib-0015] Grune T , Shringarpure R , Sitte N & Davies K (2001). Age‐related changes in protein oxidation and proteolysis in mammalian cells. J Gerontol A Biol Sci Med Sci 56, B459–467. 11682566 10.1093/gerona/56.11.b459

[tjp12512-bib-0016] Harrison DE , Strong R , Sharp ZD , Nelson JF , Astle CM , Flurkey K , Nadon NL , Wilkinson JE , Frenkel K , Carter CS , Pahor M , Javors MA , Fernandez E & Miller RA (2009). Rapamycin fed late in life extends lifespan in genetically heterogeneous mice. Nature 460, 392–395. 19587680 10.1038/nature08221 PMC2786175

[tjp12512-bib-0017] Henis‐Korenblit S , Zhang P , Hansen M , McCormick M , Lee SJ , Cary M & Kenyon C (2010). Insulin/IGF‐1 signaling mutants reprogram ER stress response regulators to promote longevity. Proc Natl Acad Sci USA 107, 9730–9735. 20460307 10.1073/pnas.1002575107 PMC2906894

[tjp12512-bib-0018] Hofmann JW , Zhao X , De Cecco M , Peterson AL , Pagliaroli L , Manivannan J , Hubbard GB , Ikeno Y , Zhang Y , Feng B , Li X , Serre T , Qi W , Van Remmen H , Miller RA , Bath KG , de Cabo R , Xu H , Neretti N & Sedivy JM (2015). Reduced expression of MYC increases longevity and enhances healthspan. Cell 160, 477–488. 25619689 10.1016/j.cell.2014.12.016 PMC4624921

[tjp12512-bib-0019] Houtkooper RH , Mouchiroud L , Ryu D , Moullan N , Katsyuba E , Knott G , Williams RW & Auwerx J (2013). Mitonuclear protein imbalance as a conserved longevity mechanism. Nature 497, 451–457. 23698443 10.1038/nature12188 PMC3663447

[tjp12512-bib-0020] Johnston‐Carey HK , Pomatto LC & Davies KJ (2015). The immunoproteasome in oxidative stress, aging, and disease. Crit Rev Biochem Mol Biol 51, 268–281. 27098648 10.3109/10409238.2016.1172554 PMC4968084

[tjp12512-bib-0021] Jovaisaite V & Auwerx J (2015). The mitochondrial unfolded protein response‐synchronizing genomes. Curr Opin Cell Biol 33, 74–81. 25543897 10.1016/j.ceb.2014.12.003 PMC4380587

[tjp12512-bib-0022] Jung T & Grune T (2012). Structure of the proteasome. Prog Mol Biol Transl Sci 109, 1–39. 22727418 10.1016/B978-0-12-397863-9.00001-8

[tjp12512-bib-0023] Kruegel U , Robison B , Dange T , Kahlert G , Delaney JR , Kotireddy S , Tsuchiya M , Tsuchiyama S , Murakami CJ , Schleit J , Sutphin G , Carr D , Tar K , Dittmar G , Kaeberlein M , Kennedy BK & Schmidt M (2011). Elevated proteasome capacity extends replicative lifespan in *Saccharomyces cerevisiae* . PLoS Genet 7, e1002253. 21931558 10.1371/journal.pgen.1002253 PMC3169524

[tjp12512-bib-0024] Labbadia J & Morimoto RI (2015). The biology of proteostasis in aging and disease. Annu Rev Biochem 84, 435–464. 25784053 10.1146/annurev-biochem-060614-033955 PMC4539002

[tjp12512-bib-0025] Lamming DW , Ye L , Sabatini DM & Baur JA (2013). Rapalogs and mTOR inhibitors as anti‐aging therapeutics. J Clin Invest 123, 980–989. 23454761 10.1172/JCI64099 PMC3582126

[tjp12512-bib-0026] Lapierre LR , Kumsta C , Sandri M , Ballabio A & Hansen M (2015). Transcriptional and epigenetic regulation of autophagy in aging. Autophagy 11, 867–880. 25836756 10.1080/15548627.2015.1034410 PMC4502732

[tjp12512-bib-0027] Lapointe J & Hekimi S (2008). Early mitochondrial dysfunction in long‐lived *Mclk1* ^+/−^ mice. J Biol Chem 283, 26217–26227. 18635541 10.1074/jbc.M803287200 PMC3258865

[tjp12512-bib-0028] Li W , Li X & Miller RA (2014). ATF4 activity: a common feature shared by many kinds of slow‐aging mice. Aging Cell 13, 1012–1018. 25156122 10.1111/acel.12264 PMC4326926

[tjp12512-bib-0029] Li W & Miller RA (2015). Elevated ATF4 function in fibroblasts and liver of slow‐aging mutant mice. J Gerontol A Biol Sci Med Sci 70, 263–272. 24691093 10.1093/gerona/glu040 PMC4351389

[tjp12512-bib-0030] Li Y & de Magalhaes JP (2013). Accelerated protein evolution analysis reveals genes and pathways associated with the evolution of mammalian longevity. Age (Dordr) 35, 301–314. 22205409 10.1007/s11357-011-9361-y PMC3592953

[tjp12512-bib-0031] Lin YF , Schulz AM , Pellegrino MW , Lu Y , Shaham S & Haynes CM (2016). Maintenance and propagation of a deleterious mitochondrial genome by the mitochondrial unfolded protein response. Nature 533, 416–419. 27135930 10.1038/nature17989 PMC4873342

[tjp12512-bib-0032] Liu HY & Pfleger CM (2013). Mutation in E1, the ubiquitin activating enzyme, reduces *Drosophila* lifespan and results in motor impairment. PLoS One 8, e32835. 23382794 10.1371/journal.pone.0032835 PMC3558519

[tjp12512-bib-0033] Luis NM , Wang L , Ortega M , Deng H , Katewa SD , Li PW , Karpac J , Jasper H & Kapahi P (2016). Intestinal IRE1 is required for increased triglyceride metabolism and longer lifespan under dietary restriction. Cell Reports 17, 1207–1216. 27783936 10.1016/j.celrep.2016.10.003 PMC5089850

[tjp12512-bib-0034] McKinnon C & Tabrizi SJ (2014). The ubiquitin‐proteasome system in neurodegeneration. Antioxid Redox Signal 21, 2302–2321. 24437518 10.1089/ars.2013.5802

[tjp12512-bib-0035] Matilainen O , Arpalahti L , Rantanen V , Hautaniemi S & Holmberg CI (2013). Insulin/IGF‐1 signaling regulates proteasome activity through the deubiquitinating enzyme UBH‐4. Cell Reports 3, 1980–1995. 23770237 10.1016/j.celrep.2013.05.012

[tjp12512-bib-0036] Merkwirth C , Jovaisaite V , Durieux J , Matilainen O , Jordan SD , Quiros PM , Steffen KK , Williams EG , Mouchiroud L , Tronnes SU , Murillo V , Wolff SC , Shaw RJ , Auwerx J & Dillin A (2016). Two conserved histone demethylases regulate mitochondrial stress‐induced longevity. Cell 165, 1209–1223. 27133168 10.1016/j.cell.2016.04.012 PMC4889222

[tjp12512-bib-0037] Min JN , Whaley RA , Sharpless NE , Lockyer P , Portbury AL & Patterson C (2008). CHIP deficiency decreases longevity, with accelerated aging phenotypes accompanied by altered protein quality control. Mol Cell Biol 28, 4018–4025. 18411298 10.1128/MCB.00296-08 PMC2423116

[tjp12512-bib-0038] Mitchell SJ , Madrigal‐Matute J , Scheibye‐Knudsen M , Fang E , Aon M , Gonzalez‐Reyes JA , Cortassa S , Kaushik S , Gonzalez‐Freire M , Patel B , Wahl D , Ali A , Calvo‐Rubio M , Buron MI , Guiterrez V , Ward TM , Palacios HH , Cai H , Frederick DW , Hine C , Broeskamp F , Habering L , Dawson J , Beasley TM , Wan J , Ikeno Y , Hubbard G , Becker KG , Zhang Y , Bohr VA , Longo DL , Navas P , Ferrucci L , Sinclair DA , Cohen P , Egan JM , Mitchell JR , Baur JA , Allison DB , Anson RM , Villalba JM , Madeo F , Cuervo AM , Pearson KJ , Ingram DK , Bernier M & de Cabo R (2016). Effects of sex, strain, and energy intake on hallmarks of aging in mice. Cell Metab 23, 1093–1112. 27304509 10.1016/j.cmet.2016.05.027 PMC4911707

[tjp12512-bib-0039] Moll L , Ben‐Gedalya T , Reuveni H & Cohen E (2016). The inhibition of IGF‐1 signaling promotes proteostasis by enhancing protein aggregation and deposition. FASEB J 30, 1656–1669. 26722006 10.1096/fj.15-281675

[tjp12512-bib-0040] Mulvey L , Sands WA , Salin K , Carr AE & Selman C (2016). Disentangling the effect of dietary restriction on mitochondrial function using recombinant inbred mice. Mol Cell Endocrinol 455, 41–53. 27597651 10.1016/j.mce.2016.09.001

[tjp12512-bib-0041] Naidoo N (2009). ER and aging‐Protein folding and the ER stress response. Ageing Res Rev 8, 150–159. 19491040 10.1016/j.arr.2009.03.001

[tjp12512-bib-0042] Novosyadlyy R , Kurshan N , Lann D , Vijayakumar A , Yakar S & LeRoith D (2008). Insulin‐like growth factor‐I protects cells from ER stress‐induced apoptosis via enhancement of the adaptive capacity of endoplasmic reticulum. Cell Death Differ 15, 1304–1317. 18437163 10.1038/cdd.2008.52

[tjp12512-bib-0043] Nuss JE , Choksi KB , DeFord JH & Papaconstantinou J (2008). Decreased enzyme activities of chaperones PDI and BiP in aged mouse livers. Biochem Biophys Res Commun 365, 355–361. 17996725 10.1016/j.bbrc.2007.10.194 PMC2238339

[tjp12512-bib-0044] O'Brien D & van Oosten‐Hawle P (2016). Regulation of cell‐non‐autonomous proteostasis in metazoans. Essays Biochem 60, 133–142. 27744329 10.1042/EBC20160006 PMC5065704

[tjp12512-bib-0045] Owusu‐Ansah E , Song W & Perrimon N (2013). Muscle mitohormesis promotes longevity via systemic repression of insulin signaling. Cell 155, 699–712. 24243023 10.1016/j.cell.2013.09.021 PMC3856681

[tjp12512-bib-0046] Page MM , Sinclair A , Robb EL , Stuart JA , Withers DJ & Selman C (2014). Fibroblasts derived from long‐lived insulin receptor substrate 1 null mice are not resistant to multiple forms of stress. Aging Cell 13, 962–964. 25059507 10.1111/acel.12255 PMC4331740

[tjp12512-bib-0047] Passtoors WM , Beekman M , Deelen J , van der Breggen R , Maier AB , Guigas B , Derhovanessian E , van Heemst D , de Craen AJ , Gunn DA , Pawelec G & Slagboom PE (2013). Gene expression analysis of mTOR pathway: association with human longevity. Aging Cell 12, 24–31. 23061800 10.1111/acel.12015

[tjp12512-bib-0048] Pharaoh G , Pulliam D , Hill S , Sataranatarajan K & Van Remmen H (2016). Ablation of the mitochondrial complex IV assembly protein Surf1 leads to increased expression of the UPR(MT) and increased resistance to oxidative stress in primary cultures of fibroblasts. Redox Biol 8, 430–438. 27208630 10.1016/j.redox.2016.05.001 PMC4878459

[tjp12512-bib-0049] Pickering AM , Lehr M & Miller RA (2015). Lifespan of mice and primates correlates with immunoproteasome expression. J Clin Invest 125, 2059–2068. 25866968 10.1172/JCI80514 PMC4463211

[tjp12512-bib-0050] Pride H , Yu Z , Sunchu B , Mochnick J , Coles A , Zhang Y , Buffenstein R , Hornsby PJ , Austad SN & Perez VI (2015). Long‐lived species have improved proteostasis compared to phylogenetically‐related shorter‐lived species. Biochem Biophys Res Commun 457, 669–675. 25615820 10.1016/j.bbrc.2015.01.046

[tjp12512-bib-0051] Rana A , Rera M & Walker DW (2013). Parkin overexpression during aging reduces proteotoxicity, alters mitochondrial dynamics, and extends lifespan. Proc Natl Acad Sci USA 110, 8638–8643. 23650379 10.1073/pnas.1216197110 PMC3666724

[tjp12512-bib-0052] Rauthan M , Ranji P , Abukar R & Pilon M (2015). A mutation in *Caenorhabditis elegans* NDUF‐7 activates the mitochondrial stress response and prolongs lifespan via ROS and CED‐4. G3 (Bethesda) 5, 1639–1648. 26038366 10.1534/g3.115.018598 PMC4528320

[tjp12512-bib-0053] Raynes R , Juarez C , Pomatto LC , Sieburth D & Davies KJ (2017). Aging and SKN‐1‐dependent loss of 20S proteasome adaptation to oxidative stress in *C. elegans* . J Gerontol A Biol Sci Med Sci 72, 143–151. 27341854 10.1093/gerona/glw093 PMC5233911

[tjp12512-bib-0054] Ristow M & Schmeisser S (2011). Extending life span by increasing oxidative stress. Free Radic Biol Med 51, 327–336. 21619928 10.1016/j.freeradbiomed.2011.05.010

[tjp12512-bib-0055] Rodriguez KA , Valentine JM , Kramer DA , Gelfond JA , Kristan DM , Nevo E & Buffenstein R (2016). Determinants of rodent longevity in the chaperone‐protein degradation network. Cell Stress Chaperones 21, 453–466. 26894765 10.1007/s12192-016-0672-x PMC4837185

[tjp12512-bib-0056] Sadighi Akha AA , Harper JM , Salmon AB , Schroeder BA , Tyra HM , Rutkowski DT & Miller RA (2011). Heightened induction of proapoptotic signals in response to endoplasmic reticulum stress in primary fibroblasts from a mouse model of longevity. J Biol Chem 286, 30344–30351. 21757703 10.1074/jbc.M111.220541 PMC3162393

[tjp12512-bib-0057] Salmon AB , Sadighi Akha AA , Buffenstein R & Miller RA (2008). Fibroblasts from naked mole‐rats are resistant to multiple forms of cell injury, but sensitive to peroxide, ultraviolet light, and endoplasmic reticulum stress. J Gerontol A Biol Sci Med Sci 63, 232–241. 18375872 10.1093/gerona/63.3.232 PMC2710579

[tjp12512-bib-0058] Salway KD , Gallagher EJ , Page MM & Stuart JA (2011a). Higher levels of heat shock proteins in longer‐lived mammals and birds. Mech Ageing Dev 132, 287–297. 21703294 10.1016/j.mad.2011.06.002

[tjp12512-bib-0059] Salway KD , Page MM , Faure PA , Burness G & Stuart JA (2011b). Enhanced protein repair and recycling are not correlated with longevity in 15 vertebrate endotherm species. Age (Dordr) 33, 33–47. 20567926 10.1007/s11357-010-9157-5 PMC3063641

[tjp12512-bib-0060] Selman C (2014). Dietary restriction and the pursuit of effective mimetics. Proc Nutr Soc 73, 260–270. 24411076 10.1017/S0029665113003832

[tjp12512-bib-0061] Selman C , Lingard S , Choudhury AI , Batterham RL , Claret M , Clements M , Ramadani F , Okkenhaug K , Schuster E , Blanc E , Piper MD , Al‐Qassab H , Speakman JR , Carmignac D , Robinson IC , Thornton JM , Gems D , Partridge L & Withers DJ (2008). Evidence for lifespan extension and delayed age‐related biomarkers in insulin receptor substrate 1 null mice. FASEB J 22, 807–818. 17928362 10.1096/fj.07-9261com

[tjp12512-bib-0062] Selman C , Tullet JM , Wieser D , Irvine E , Lingard SJ , Choudhury AI , Claret M , Al‐Qassab H , Carmignac D , Ramadani F , Woods A , Robinson IC , Schuster E , Batterham RL , Kozma SC , Thomas G , Carling D , Okkenhaug K , Thornton JM , Partridge L , Gems D & Withers DJ (2009). Ribosomal protein S6 kinase 1 signaling regulates mammalian life span. Science 326, 140–144. 19797661 10.1126/science.1177221 PMC4954603

[tjp12512-bib-0063] Shore DE , Carr CE & Ruvkun G (2012). Induction of cytoprotective pathways is central to the extension of lifespan conferred by multiple longevity pathways. PLoS Genet 8, e1002792. 22829775 10.1371/journal.pgen.1002792 PMC3400582

[tjp12512-bib-0064] Swindell WR , Masternak MM , Kopchick JJ , Conover CA , Bartke A & Miller RA (2009). Endocrine regulation of heat shock protein mRNA levels in long‐lived dwarf mice. Mech Ageing Dev 130, 393–400. 19428459 10.1016/j.mad.2009.03.004 PMC2718793

[tjp12512-bib-0065] Tawo R , Pokrzywa W , Kevei E , Akyuz ME , Balaji V , Adrian S , Hohfeld J & Hoppe T (2017). The ubiquitin ligase CHIP integrates proteostasis and aging by regulation of insulin receptor turnover. Cell 169, 470–482 e413. 28431247 10.1016/j.cell.2017.04.003 PMC5406386

[tjp12512-bib-0066] Taylor RC & Dillin A (2013). XBP‐1 is a cell‐nonautonomous regulator of stress resistance and longevity. Cell 153, 1435–1447. 23791175 10.1016/j.cell.2013.05.042 PMC4771415

[tjp12512-bib-0067] Tonoki A , Kuranaga E , Tomioka T , Hamazaki J , Murata S , Tanaka K & Miura M (2009). Genetic evidence linking age‐dependent attenuation of the 26S proteasome with the aging process. Mol Cell Biol 29, 1095–1106. 19075009 10.1128/MCB.01227-08 PMC2643801

[tjp12512-bib-0068] Vilchez D , Saez I & Dillin A (2014). The role of protein clearance mechanisms in organismal ageing and age‐related diseases. Nat Commun 5, 5659. 25482515 10.1038/ncomms6659

[tjp12512-bib-0069] Zhang HM , Fu J , Hamilton R , Diaz V & Zhang Y (2015). The mammalian target of rapamycin modulates the immunoproteasome system in the heart. J Mol Cell Cardiol 86, 158–167. 26239133 10.1016/j.yjmcc.2015.07.027

